# Mitochondrial mutation status, symptom prevalence, and neuroimaging characteristics in MELAS: a systematic review and meta analysis

**DOI:** 10.1186/s13023-026-04448-6

**Published:** 2026-06-10

**Authors:** Yanyan Li, Siying Han, Daiyun Huang, Jiayi Chen, Siying Qu, Jinxin Gu, Zhuoyan Xu, Lei Fu, Xiaoyu Tang, Yixue Qiao

**Affiliations:** 1 Wisdom Lake Academy of Pharmacy, Xi’an Jiaotong-Liverpool University, Suzhou, China; 2 Institute of Population Health, Faculty of Health & Life Sciences, University of Liverpool, Waterhouse Building, Liverpool, England; 3 Department of Biostatistics, Boston University, Boston, MA USA

**Keywords:** MELAS, m.3243A > G mutation, Symptom prevalence, Neuroimaging, Meta analysis, Therapeutic target prediction

## Abstract

**Background:**

Mitochondrial encephalomyopathy, lactic acidosis, and stroke-like episodes (MELAS) is a rare mitochondrial disorder characterized by a wide range of systemic manifestations. MELAS is challenging to diagnose in its early stages and currently lacks targeted treatments. This systematic review and meta-analysis aim to validate the mitochondrial 3243A > G (m.3243A > G) mutation status, symptom prevalence, and neuroimaging characteristics in patients with MELAS. The review also seeks to consolidate and clarify the pathogenic pathways of MELAS while pinpointing potential therapeutic targets.

**Main body:**

We searched four databases for relevant studies. Study selection, data extraction, and quality assessment using the JBI (Joanna Briggs Institute) scale were performed by two reviewers. We conducted meta-analyses to estimate the pooled proportions for genetic, symptomatic, and imaging features using the random-effects generalized linear mixed model. In addition, seven MELAS-related pathways were researched, and their gene information was expanded through computational retrieval from the Kyoto Encyclopedia of Genes and Genomes (KEGG) and Reactome databases. A total of 13 studies involving 988 subjects were included, covering one mutant gene (m.3243A > G), ten clinical symptoms, and four imaging features. The meta-analyses showed a pooled m.3243A > G mutation prevalence of 78% (95% CI: 68%, 86%). The most frequent clinical manifestations were stroke-like episodes (99%; 95% CI: 29%, 100%), seizures (89%; 95% CI: 65%, 97%), and elevated lactate (81%; 95% CI: 50%, 95%). The temporal lobe was the most commonly affected brain region on imaging (87%; 95% CI: 13%, 100%). Pathway analysis revealed mitophagy/autophagy, ROS generation, and energy metabolism as core mechanisms in MELAS, with MNRR1, L-arginine, PI3K-AKT-mTOR, and ETC as potential therapeutic targets.

**Conclusion:**

This study collects evidence for the confirmatory diagnosis of MELAS, integrating its genetic, clinical, and neuroimaging spectra. Furthermore, by elucidating the core pathological network, our work provides a potential framework for developing multi-targeted therapeutic strategies.

**Supplementary Information:**

The online version contains supplementary material available at 10.1186/s13023-026-04448-6.

## Background

Mitochondrial encephalomyopathy, lactic acidosis, and stroke-like episodes (MELAS) syndrome is one of the most common maternally inherited mitochondrial disorders, primarily caused by mutations in mitochondrial DNA (mtDNA). Its characteristic clinical manifestations include stroke-like episodes, seizures, headaches, vomiting, cognitive impairment, and muscle weakness [1]. MELAS predominantly affects children and adolescents, and its highly heterogeneous and non-specific presentations can easily be confused with other neurological conditions such as ischemic stroke, or epilepsy. Currently, the diagnosis of MELAS relies on an evaluation of genetic testing, clinical symptoms, biochemical indicators, and neuroimaging findings [2]. This diagnostic complexity often leads to misdiagnosis or delayed diagnosis, resulting in missed opportunities for early intervention and imposing a substantial burden on patients and their families [3, 4].

Although numerous studies have described different aspects of MELAS, many of them are limited by small sample sizes or focus on a single dimension, leading to uncertainty in conclusions. Considerable variation exists across studies regarding the reported incidence of key diagnostic features, and there is a lack of systematic and high-quality evidence synthesis to confirm their overall diagnostic utility. Meanwhile, despite progress in understanding the disease, effective treatments remain limited. Current therapies primarily involve supportive approaches, and a phase II clinical trial of arginine is currently underway [5]. Therefore, further investigation into the pathogenesis of MELAS is essential for developing targeted and effective treatment strategies.

Systematic reviews and meta-analyses, as essential tools in evidence-based medicine, synthesize results from multiple independent studies to provide more reliable and comprehensive conclusions [6]. Therefore, this study adopts rigorous systematic review and meta-analysis methods to integrate existing evidence, quantitatively confirming the prevalence of the m.3243A > G mutation, the frequency of core clinical manifestations, and the distribution of neuroimaging involvement across different brain lobes in patients with MELAS syndrome. This study will also systematically summarize and analyze the pathogenic network of MELAS and predict potential therapeutic targets for drug design. This work will provide a clearer and evidence-based diagnostic probability framework and management strategy for clinicians, thereby enhancing early recognition and accurate diagnosis of the disease and ultimately improving patient outcomes. This study was registered in PROSPERO (ID: 1159415).

## Methods

### Retrieval strategy

This study was conducted in accordance with the Preferred Reporting Items for Systematic Reviews and Meta-Analyses (PRISMA) guidelines. A systematic search was performed in four English-language electronic databases: PubMed, Embase, the Cochrane Library, and Web of Science (SCI-E), covering the period from the inception of each database to August 15, 2025. To minimize the risk of missing relevant studies, the reference lists of the included articles were also manually screened. The search strategy was constructed using a combination of controlled vocabulary terms (MeSH/Emtree) and free-text keywords. Based on the research objectives, the primary search concepts focused on “MELAS syndrome” and related diagnostic indicators such as “gene mutation”. The specific search strategy is shown in Table S1.

### Inclusion and exclusion criteria

Studies were included if they met the following inclusion criteria: (1) study type, encompassing observational studies (cross-sectional, cohort, case-control or randomized controlled trial) reporting the prevalence of the m.3243A > G mutation, clinical manifestations, or neuroimaging characteristics in patients with MELAS syndrome; (2) study population, consisting of patients diagnosed with MELAS syndrome based on both clinical presentation and genetic confirmation; and (3) data completeness, with studies providing extractable or convertible data relevant to the target outcomes. Studies were excluded if they met any of the following criteria: (1) duplicate publications; (2) case reports, review articles, conference abstracts, editorials, or other non-original research; (3) lack of full-text availability or incomplete/non-usable data; or (4) publication in languages other than English.

### Literature screening, data extraction, and quality assessment

According to the predefined inclusion and exclusion criteria, two researchers independently conducted literature screening and data extraction. Duplicate records were first removed using reference management software (EndNote X9), followed by an initial screening of titles and abstracts to exclude clearly irrelevant studies. Full texts of potentially eligible studies were then retrieved and carefully reviewed to determine final inclusion. Any disagreements during the screening process were resolved through discussion or, when necessary, consultation with a third researcher.

Using a pre-designed data extraction form, the two researchers independently extracted the relevant data, which were subsequently cross-checked for accuracy. Extracted information primarily included: (1) basic study characteristics, such as first author, publication year, country, and study type; and (2) data for outcomes of interest: ① m.3243A > G mutation: number of patients positive for the m.3243A > G mutation and the total number of MELAS patients tested; ② Clinical manifestation: number of patients exhibiting specific clinical symptoms and the total number of MELAS patients; ③ Neuroimaging manifestation: number of patients showing involvement of the parietal, frontal, temporal, or occipital lobes, or basal ganglia on brain MRI divided and the total number of MELAS patients who underwent neuroimaging.

### Statistical analysis

We calculated the proportions along with their corresponding 95% confidence intervals (CIs) of the following features of MELAS patients reported in the included studies: the presence of the m.3243A > G mutation rate, specific clinical symptoms mentioned above, and affected brain regions. Using random-effects generalized linear mixed models with a logit link, we conducted meta-analyses to estimate the pooled proportions for each of these categories. Heterogeneity between studies was assessed by likelihood ratio test statistics and quantified by the I² statistic. Substantial heterogeneity was defined as a likelihood ratio test p-value < 0.05 and I² > 50%. We further conducted subgroup analyses to explore potential sources of heterogeneity. Age-based subgroups were defined using the median onset age reported in each study, or the mean when the median was unavailable, and dichotomized using a uniform cutoff of 18 years (≤ 18 vs. >18). Sex-based subgroups were defined using a 50% cutoff for the proportion of female participants (≤ 50% vs. >50%). Finally, we generated forest plots to visualize the proportions and 95% CIs for individual studies, as well as the pooled overall proportion for each feature. Meta-analyses were not conducted if the number of studies was less than 3.

### Risk of bias

The risk of bias for included studies was assessed independently by two authors using the Joanna Briggs Institute (JBI) scale [7]. Discrepancies were resolved through discussion with team members. A total of nine aspects were assessed, including: sample frame, sampling method, sample size, characteristics of participants and setting, coverage of data analysis, identification of condition, reliability of condition measurement, statistical analysis, and response rate and its adjustment. The assessment results were classified as low risk, moderate risk, and high risk.

## Results

### Literature identified

**Fig. 1:**
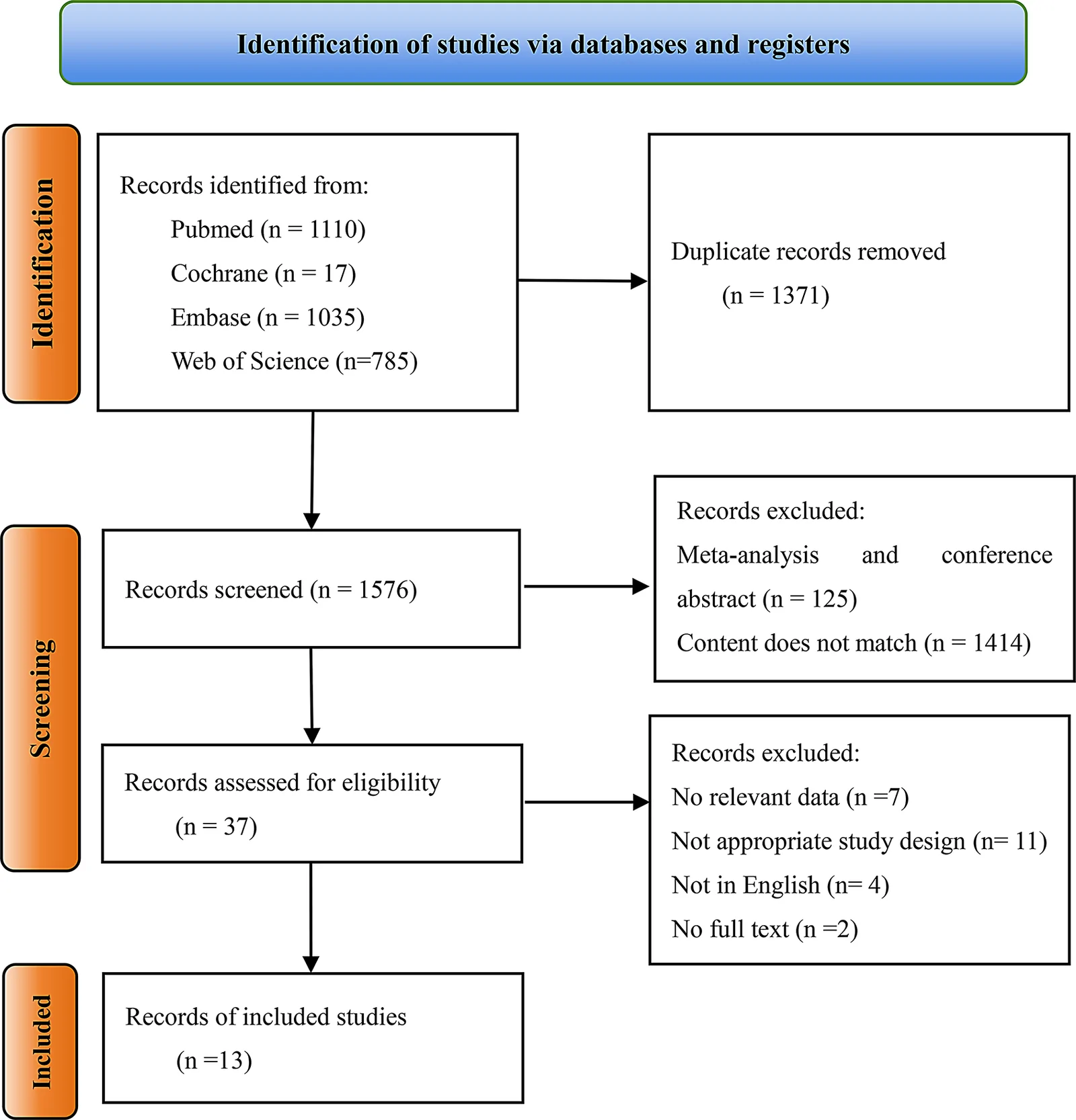
Study selection

The initial search retrieved 2,947 records from PubMed, Cochrane Library, Embase, and Web of Science. After removing 1,371 duplicates, 1,576 articles remained for title and abstract screening. Following the exclusion of 1,539 ineligible studies, 37 articles underwent full-text review, of which 24 were excluded for not meeting the inclusion criteria, resulting in 13 studies being included in the meta-analysis. The literature screening process was summarized in Fig. 1, and the characteristics of the included studies were summarized in Table S2 [1–3, 8–17]. These 13 studies encompassed a total of 988 patients clinically and genetically diagnosed with MELAS syndrome, with publication years ranging from 1991 to 2024 and study populations spanning Asia, Europe, and the Americas. Among them, 8 studies reported the prevalence of the m.3243A > G mutation, 10 reported at least one clinical manifestation, and 4 reported the incidence of at least one neuroimaging feature.

### m.3243A > G mutation and MELAS

**Fig. 2:**
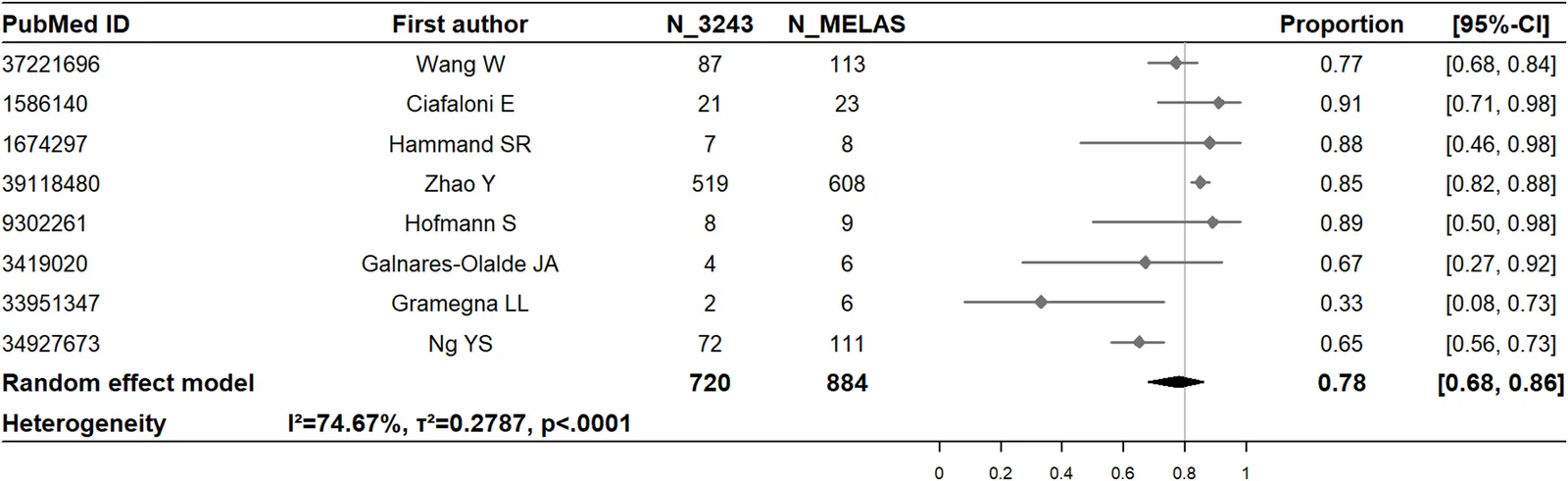
Forest Plot of meta-analysis for m.3243A > G in MELAS (random-effects generalized linear mixed model). N_3243 = number of patients with m.3243A > G mutation, N_MELAS = number of MELAS patients

The meta-analysis of the m.3243A > G mutation revealed an estimated pooled prevalence of 78% (95% CI: 68%, 86%) (Fig. 2). In subgroup analyses (Table S3 and Table S4), heterogeneity decreased in the ≥ 18 years onset age group and the > 50% female proportion group. Pooled estimates in subgroups were similar to the overall effect.

### Symptoms and MELAS

Except for ataxia and elevated lactate, significant heterogeneity was observed across studies for all other clinical manifestations (I² > 50%, *p* < 0.01). The combined prevalence of each symptom, in descending order, is presented in Table 1 and Figure S1-S3. Based on the point estimates, the top three clinical manifestations were stroke-like episodes (99%; 95% CI: 29%, 100%), seizures (89%; 95% CI: 65%, 97%), and elevated lactate (81%; 95% CI: 50%, 95%).

For clinical symptoms, subgroup analyses (Table S3 and Table S4) showed that heterogeneity was reduced for hearing loss, migraine, myopathy, or seizure in the > 18-year onset age subgroup, while seizure showed reduced heterogeneity in the ≤ 18-year subgroup. In sex-stratified analyses, heterogeneity decreased for hearing loss in both subgroups and was lower for seizure in the ≤ 50% female proportion subgroup. Hence, gender and onset age are potential sources of heterogeneity. Most pooled estimates were consistent with the overall analysis, whereas lower proportions of seizure and hearing loss were observed in the ≤ 50% female proportion subgroup, and a lower proportion of MERRF and higher proportion of migraine were observed in the > 18-year onset age subgroup.

**Table 1:** Summary of pooled proportions for key symptoms

Symptoms	Pooled Proportion (95% CI)	Heterogeneity		
		I²	τ²	*p*
Stroke	0.99 (0.29, 1.00)	87.80%	9.24	0.002
Seizure	0.89 (0.65, 0.97)	95.82%	2.99	< 0.001
Lactate elevated	0.81 (0.50, 0.95)	82.48%	1.24	< 0.001
MERRF*	0.75 (0.64, 0.84)	4.26%	0.02	0.362
Blindness	0.70 (0.29, 0.93)	92.10%	2.39	< 0.001
Diabetes	0.62 (0.17, 0.93)	87.00%	3.60	< 0.001
Hearing loss	0.51 (0.30, 0.72)	92.78%	1.31	< 0.001
Migraine	0.45 (0.30, 0.61)	79.92%	0.31	< 0.001
Myopathy	0.41 (0.23, 0.62)	0.00%	0.00	0.196
Ataxia	0.14 (0.10, 0.21)	0.00%	0.00	0.423

* MERRF, Myoclonic Epilepsy with Ragged-Red Fibers

### Neuroimaging (Brain MRI) and MELAS

Substantial heterogeneity across studies was found in the analysis for the temporal lobe and basal ganglion (I² > 50%, *p* < 0.05). The results are presented in descending order of prevalence in Table 2. Based on the point estimate of the combined proportion, the temporal lobe is the most relevant with MELAS (87%; 95% CI: 13%, 100%). Subgroup analyses (Table S3 and Table S4) showed that heterogeneity remained unchanged for occipital and parietal lesions but increased for temporal lesions. Compared with the overall combined proportions, Temporal and parietal involvement were higher in the > 18-year subgroup, while occipital involvement showed minimal change (< 0.02). Hence, the onset age may not be a source of heterogeneity in brain region analysis. Due to the data limitation, subgroup analysis by gender were not conducted.

**Table 2:** Summary of pooled proportions for brain locations

Brain regions affected by SLE*	Pooled Proportion (95% CI)	Heterogeneity		
		I²	τ²	*p*
Temporal lobe	0.87 (0.13, 1.00)	90.58%	8.70	< 0.001
Occipital lobe	0.48 (0.38, 0.59)	0.00%	0.00	0.773
Parietal lobe	0.44 (0.34, 0.54)	0.00%	0.00	0.177
Basal ganglion	0.14 (0.04, 0.42)	61.24%	0.93	0.023

* SLE, Stroke-like Episodes

### Assessment of bias

A total of 13 studies were included in the analysis. Using the JBI scale for risk of bias assessment, three studies (23%) were rated as low risk, while five (38%) each were rated as moderate and high risk (Table S5). The domains most frequently contributing to bias were the adequacy of the sample size, the sufficiency of data analysis coverage, and the representativeness of the sampling frame. These findings are not unexpected given the rarity of MELAS and the mechanistic focus of several studies, reflecting the inherent practical challenges in minimizing bias in rare disease research.

### Pathway and MELAS

The pathogenesis of MELAS involves a complex interplay of multiple interconnected cellular pathways centered around mitochondrial mutation dysfunction. Key pathways include MNRR1 [18], which acts as a bi-organellar regulator to induce mitochondrial biogenesis, UPRmt, and autophagy, thereby rescuing cellular function. The PI3K-Akt-mTORC1 [19] axis is constitutively activated, promoting metabolic rewiring and lactate production, while AMPK-mediated signaling [20] reveals metabolic inflexibility, impairing the switch to fatty acid oxidation and reinforcing glycolytic dependency. ATAD3B [21] facilitates PINK1-independent mitophagy to clear damaged mtDNA, and ROS-sensitive microRNA-9/9∗ [22], induced via NF-κB signaling, disrupts mitochondrial tRNA modification. Additionally, dysregulation of the glutamate [23] and arginine [24] biosynthesis pathways highlights metabolic imbalances, with glutamate accumulation and arginine deficiency further contributing to disease progression. Many of these pathways are ROS-sensitive and exhibit significant crosstalk, particularly in processes involving autophagy/mitophagy, and metabolic adaptation, underscoring their collective role in MELAS pathophysiology and potential as therapeutic targets.

Pathway and gene information relevant to MELAS was expanded through systematic retrieval from the Kyoto Encyclopedia of Genes and Genomes (KEGG; https://www.kegg.jp) and Reactome (https://reactome.org/) databases. Using the R Bioconductor packages KEGGREST and reactome.db, we programmatically accessed curated pathway annotations. KEGGREST provides a client interface to the KEGG REST API, and we employed its keggGet function to retrieve detailed pathway records. reactome.db supplies precompiled annotation maps derived from Reactome, and we used the reactomePATHID2NAME mapping to link Reactome pathway identifiers to Entrez Gene identifiers.

From KEGG, we specifically retrieved the following human pathways using their official identifiers: PI3K-Akt signaling pathway (hsa04151), AMPK signaling pathway (hsa04152), Glutamatergic synapse (hsa04724), and Arginine biosynthesis (hsa00220). For the gene ATAD3B, we obtained the autophagy-related pathway hsa04140 from KEGG and complemented it with the mitophagy pathway from Reactome (R-HSA-5205647). For the ROS-sensitive microRNA-9/9*, we retrieved the NF-κB signaling pathway from KEGG (hsa04064) and the mitochondrial tRNA modification pathway from Reactome (R-HSA-6787450). In the case of MNRR1 (also known as CHCHD2), no associated pathways were found in KEGG; however, two mitochondrial-related pathways in Reactome that include MNRR1 were identified: mitochondrial protein import (R-HSA-1268020) and mitochondrial protein degradation (R-HSA-9837999).

Based on the shared gene analysis across the MELAS-related pathways (Table S6), several key functional groups and nodal genes emerge, highlighting the interconnectedness of the cellular processes involved: (1) Mitochondrial Protein Import and Mitophagy: The mitochondrial protein import and degradation pathways share 14 genes, primarily Translocase of the Outer Membrane (TOM) complex subunits. Mitophagy also shares eight genes with the import pathway, physically linking protein import to mitophagy initiation. (2) Autophagy and Signaling Crosstalk: Core autophagy genes are shared between general autophagy and mitophagy. Significant crosstalk exists, with key signaling genes (AKT1, MTOR, ULK1) shared across PI3K, AMPK, and autophagy pathways. Genes like BCL2 and TRAF6 link NF-κB signaling directly to autophagy. (3) Metabolic Hubs and Key Integrators: Glutamate and arginine biosynthesis pathways share genes like GLS, underscoring their metabolic link. The glutamate pathway extensively overlaps with the PI3K pathway. PRKACA is a critical integrator, shared across glutamate signaling, autophagy, and mitochondrial degradation. For arginine metabolism, NOS3, ARG2, and GLUD1 connect to PI3K signaling and mitochondrial processes.

## Discussion

### Rationale for the inclusion and exclusion criteria

Although MELAS is widely reported clinically, most published papers are case reports, case series, or narrative reviews lacking extractable prevalence data for quantitative meta‑analysis. We established strict inclusion criteria to ensure rigor: restricting included studies to observational designs (cross-sectional, cohort, case-control) and RCTs is justified as they provide the systematic, standardized quantitative data on MELAS; requiring MELAS diagnosis via both clinical presentation and genetic confirmation ensures sample homogeneity and accuracy by minimizing misdiagnosis and bias from mimicking disorders; and the data completeness criterion (extractable/convertible target outcome data) is essential to effectively pool and quantify MELAS-related prevalence estimates and ensure all included studies contribute meaningfully to the synthesis. We excluded studies involving patients classified as suspected MELAS or those with MELAS/Leigh syndrome overlap. This exclusion was intended to ensure a more homogeneous study population with clearly defined MELAS diagnostic criteria, thereby enhancing the internal validity of the pooled estimates.

### The relationship between m.3243A > G mutation and MELAS

This study confirmed that the m.3243A > G mutation represents the primary genetic basis of MELAS. This finding is highly consistent with previous studies [1, 2–4, 9, 10, 14–16], further confirming the central role of the m.3243A > G mutation in MELAS pathogenesis. The high detection rate of this specific mutation establishes it as a pivotal molecular diagnostic marker for MELAS syndrome, providing decisive value in confirming the genetic diagnosis. Although m.3243A > G mutation is the predominant mutation in MELAS, approximately 22% of patients tested negative, suggesting the involvement of other pathogenic mtDNA or nuclear DNA mutations, such as m. 3271T > C, m. 3252 A > G and m. 13,513 G > A [25, 26]. Therefore, in clinical genetic diagnosis, if MELAS is strongly suspected but m.3243A > G testing is negative, further expanded genetic testing is warranted to avoid missed diagnoses.

### The mechanisms underlying the incidence of various symptoms in MELAS patients

This study found that stroke-like episodes, seizures, and elevated lactate were the top three common symptoms. The predominant m.3243A > G mutation in the mitochondrial tRNA-Leu (UUR) impairs respiratory chain proteins synthesis, particularly affecting Complex I assembly and function [27, 28]. This disrupts oxidative phosphorylation, causing cellular energy deficiency and a shift to anaerobic metabolism, leading to lactate accumulation [29]. In cerebrovascular endothelial cells, this energy crisis triggers microvascular dysfunction, blood-brain barrier disruption, and local ischemia. In neurons, it results in energy failure, excessive glutamate release, and calcium influx, culminating in cell death [30, 31]. These processes collectively cause the characteristic stroke-like episodes. Neuronal energy failure also destabilizes membrane potentials and impairs glutamate reuptake, leading to hyperexcitability and NMDA receptor overstimulation, which triggers seizures [32]. Other manifestations further reflect the multisystem involvement of MELAS.

### Correlation between neuroimaging (brain MRI) findings and MELAS

Brain MRI abnormalities in MELAS most commonly involve the temporal lobe. This posterior region supports high-order functions like sensory integration, visual processing, language, and memory, characterized by exceptionally high synaptic density and sustained neural activity. Consequently, their resting-state energy consumption significantly exceeds that of other brain areas. This high metabolic demand, coupled with potentially limited vasodilatory reserve in posterior vascular networks, creates an imbalance that predisposes these regions to energy failure. Notably, while respiratory chain defects occur in cerebral microvessels, the oxidative phosphorylation deficiency is more severe in neurons—particularly inhibitory interneurons—than in the microvasculature [16]. This challenges the traditional “vasculogenic” hypothesis and supports the view that stroke-like episodes are primarily driven by refractory epileptic activity and neuronal energy failure [16, 33], similar to patterns in POLG-related encephalopathy [33]. This distribution of MELAS lesions serves as a key imaging marker for differentiating MELAS from ischemic stroke [3, 16]. These features reflect mitochondrial energy failure and neurovascular unit dysfunction, making neuroimaging crucial not only for diagnosis but also for monitoring disease activity and treatment response.

### Therapeutic target prediction

**Fig. 3:**
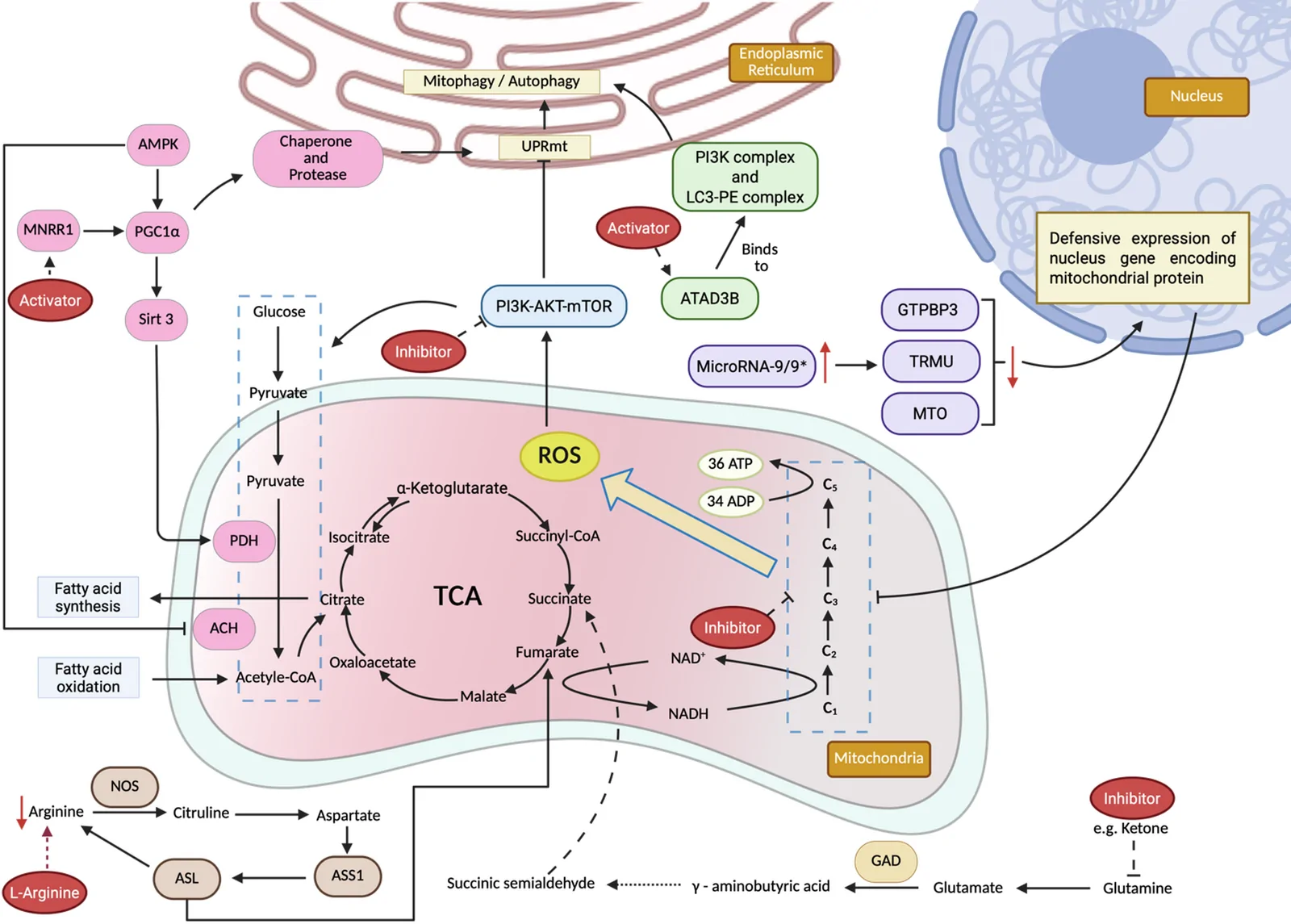
Schematic representation of the integrated cellular network implicated in MELAS pathogenesis. The diagram highlights three key interconnected mechanisms including mitophagy/autophagy, ROS production, and Glucose/Amino acid/Fatty acid metabolic pathways. Central subcellular organelles include mitochondria, endoplasmic reticulum, and nucleus. Potential drug targets and active molecules include MNRR1 (activator), arginine derived nitric oxide (L-arginine), PI3K-AKT-mTOR (inhibitor), mitochondrial electron respiration chain (inhibitor), Glutamine synthesis (inhibitor). Black arrows represent the positive regulatory or catalytic relationships. Inhibition symbols denote the suppression of a molecule’s activity by another component, clearly reflecting the dynamic balance of the biological pathways. Red arrows represent up/down regulations of the protein/genes in MELAS vs. control

Based on the pathway analysis (Fig. 3), three key interconnected mechanisms included in MELAS mitophagy/autophagy, ROS production, and Glucose/Amino acid/Fatty acid metabolic pathways. Central subcellular organelles include mitochondria, endoplasmic reticulum, and nucleus. Potential drug targets and active molecules include: (1) MNRR1 [18] serves as a key activator target. This protein plays a crucial role in regulating mitochondrial function and cellular energy metabolism, making it a promising candidate for developing activating agents that can enhance mitochondrial activity and mitigate the energy deficiency characteristic of MELAS; (2) L-arginine [24], a naturally occurring amino acid, acts as the precursor for nitric oxide (NO) synthesis. Exogenous supplementation of L-arginine effectively relieves MELAS symptoms because nitric oxide is essential for maintaining vascular homeostasis and improving blood flow to tissues, which is critical for addressing the stroke-like episodes and tissue hypoxia often seen in MELAS patients. (3) the PI3K-AKT-mTOR signaling pathway [19] is identified as an inhibitory target. This pathway is closely linked to cell growth, metabolism, and survival; aberrant activation of this pathway in MELAS may contribute to mitochondrial dysfunction. Inhibitors targeting this pathway could help restore normal cellular signaling and alleviate disease symptoms. (4) The mitochondrial electron transport chain (ETC), which is responsible for generating cellular energy (ATP), is another key inhibitory target. In MELAS, the ETC is often impaired, leading to reduced ATP production and increased reactive oxygen species (ROS) accumulation. Inhibitors that modulate ETC function could help correct mitochondrial dysfunction and reduce oxidative stress.

### Associations between pathway analysis and meta analysis results

The integrated cellular network underlying MELAS pathogenesis is closely linked to Meta-analysis findings: The meta-analysis indicated that the m.3243A > G mutation, with a pooled prevalence of 78% (95% CI: 68%–86%), is the core genetic defect in MELAS. This mutation occurs on the tRNA of complex I in the mitochondrial electron transport chain, which disrupts mitochondrial function, impairs the respiratory chain, dysregulates reactive oxygen species (ROS) production, and thereby compromises mitophagy and autophagy [27, 28]. The most frequent clinical manifestations, stroke-like episodes (99%), seizures (89%), and elevated lactate (81%), result from these dysregulations: Elevated lactate results from impaired mitochondrial function (which hinders aerobic glucose metabolism) and disrupted glucose metabolism, leading the body to rely on anaerobic glycolysis that produces excessive lactate, causing elevated lactate levels [29]; Excessive ROS can cause oxidative stress, which damages neurons, impairs signal transmission, and triggers neuroinflammation, all of which contribute to the occurrence of neurological symptoms (episodes and seizures); Dysregulated amino acid metabolism disrupts the balance of neurotransmitters, such as glutamate, GABA, affects neuronal excitability, and further exacerbates neurological discomfort [23]. The temporal lobe (most commonly affected on imaging, 87%) is highly dependent on mitochondrial energy compared to other brain areas, so its abnormalities reflect the network’s dysfunctions.

### Limitations

There are several limitations of this meta-analysis. First, the included studies exhibited variations in sample sources, diagnostic criteria, and assessment standards, leading to significant heterogeneity in most outcome indicators. We did subgroup analyses to evaluate the source of heterogeneity. However, due to the limited data reported in the original articles, there are not enough studies to conduct comprehensive subgroup analyses to explore all potential sources of heterogeneity. Second, the observational study design itself carries risks of selection and information bias. Third, the literature search was restricted to English-language publications, although critical to avoiding misinterpretation and ensuring data consistency, this may introduce language and publication bias, and our findings may be more applicable to populations represented in English-language literature. Additionally, the limited number of available studies for certain indicators (such as neuroimaging features) resulted in wide confidence intervals for some pooled estimates, which may reduce the stability of the findings and limit the potential for in-depth subgroup analyses. This may limit the utility of the results for early or atypical diagnosis. After subgrouping, the number of studies in each category was further reduced, with most subgroups including only three to four studies. This reduction may have affected the reliability of heterogeneity measures and led to instability in subgroup-specific estimates. The estimates in subgroups should be interpreted with caution. Fourth, this meta‑analysis synthesizes well‑established MELAS features (e.g., m.3243A > G predominance, stroke‑like episodes, seizures, lactic acidosis, temporal lobe involvement). While pooled prevalence estimates support confirmatory evidence, our results do not substantially improve current phenotype‑genotype understanding or diagnostic approaches, making this study largely confirmatory rather than discovery‑driven.

## Conclusion

This meta-analysis of 13 studies encompassing 988 patients confirmed that the m.3243A > G is the primary genetic cause of MELAS syndrome. The most common clinical manifestations were stroke-like episodes, elevated lactate, and seizures. Brain MRI most frequently revealed involvement of the temporal lobe. These findings provide a clear and evidence-based diagnostic profile to support the accurate identification of MELAS. Pathway analysis identified mitophagy/autophagy, ROS production, and metabolic pathways as core mechanisms in MELAS, with MNRR1, L-arginine, PI3K-AKT-mTOR, and ETC as promising therapeutic targets; meta-analysis confirmed that the prevalent m.3243A > G mutation impairs mitochondrial function, elevates ROS and lactate, and leads to stroke-like episodes, seizures, and predominant temporal lobe injury, providing integrated insights into MELAS pathogenesis and intervention strategies.

## Supplementary Information

Below is the link to the electronic supplementary material.

Supplementary Material 1

## Data Availability

All data generated or analysed during this study are included in this published article and its supplementary information files.

## Abbreviations

CIs: Confidence Intervals
JBI: Joanna Briggs Institute
KEGG: Kyoto Encyclopedia of Genes and Genomes
MELAS: Mitochondrial Encephalomyopathy, Lactic Acidosis, and Stroke-like Episodes
m.3243A > G: Mitochondrial 3243A > G
mtDNA: Mitochondrial DNA
OXPHOS: Oxidative Phosphorylation
ROS: Reactive Oxygen Species
TOM: Translocase of the Outer Membrane
