# Supplementary material for: Mitochondrial mutation status, symptom prevalence, and neuroimaging characteristics in MELAS: a systematic review and meta analysis
Source: Orphanet J Rare Dis. 2026 Jun 10;21:283. doi: 10.1186/s13023-026-04448-6 (PMC13487938; doi:10.1186/s13023-026-04448-6)
Supplement: Supplementary file 1 [file 13023_2026_4448_MOESM1_ESM.docx]

**Supplementary Information**

**Figure S1. Forest Plot of Meta-analysis for Symptoms**

**Figure S2. Forest Plot of Meta-analysis for Symptoms** Cont.

**Figure S3. Forest Plot of Meta-analysis for SLE Affected Regions**

**Table S1 Search Strategy**

| **Database** | **Search Strategy** |
| --- | --- |
| **PubMed** | ((("Mitochondria"[Mesh]) OR (Mitochondri[Title/Abstract])) AND ((((((("MELAS Syndrome"[Mesh]) OR (MELAS[Title/Abstract])) OR (Mitochondrial Myopathy, Lactic Acidosis, Stroke-Like Episode)) OR (Myopathy, Mitochondrial-Encephalopathy-Lactic Acidosis-Stroke)) OR (Mitochondrial Encephalomyopathy, Lactic Acidosis, AND Stroke-Like Episodes)) OR (Mitochondrial Myopathy, Encephalopathy, Lactic Acidosis, AND Stroke-Like Episodes)) OR (Encephalomyopathy, Mitochondrial, Lactic Acidosis, AND Stroke-like episodes))) AND (((((("Point Mutation"[Mesh]) OR ("Mutation"[Mesh])) OR (Mutation)) OR (Variant)) OR (Polymorphism)) OR (((mtDNA) AND (Base)) AND (((Substit) OR (Insert)) OR (Delet)))) AND english[la] AND "humans"[MeSH] |
| **Cochrane** | ((MeSH descriptor: [Mitochondria] explode all trees) OR (Mitochondri*:ti,ab,kw))AND((MeSH descriptor: [MELAS Syndrome] explode all trees) OR(MELAS:ti,ab,kw) OR(Mitochondrial Myopathy, Lactic Acidosis, Stroke-Like Episode:ti,ab,kw) OR (Myopathy, Mitochondrial-Encephalopathy-Lactic Acidosis-Stroke:ti,ab,kw) OR(Mitochondrial Encephalomyopathy, Lactic Acidosis, and Stroke-Like Episode:ti,ab,kw) OR (Mitochondrial Myopathy, Encephalopathy, Lactic Acidosis, And Stroke-Like Episode:ti,ab,kw) OR (Encephalomyopathy, Mitochondrial, Lactic Acidosis, and Stroke-like episodes:ti,ab,kw))AND( (MeSH descriptor: [Mutation] explode all trees) OR (MeSH descriptor: [Point Mutation] explode all trees) OR(Mutation:ti,ab,kw) OR (Variant*:ti,ab,kw) OR (Polymorphism*:ti,ab,kw) OR (Substitut*:ti,ab,kw) OR(Insert*:ti,ab,kw) OR (Delet*:ti,ab,kw) OR ((mtDNA:ti,ab,kw) AND (Base:ti,ab,kw) AND ((Substitut*:ti,ab,kw) OR (Insert*:ti,ab,kw) OR (Delet*:ti,ab,kw)))) |
| **Embase** | ( mitochondri*:ab,ti AND ( melas:ab,ti OR "mitochondrial myopathy, lactic acidosis, stroke like episode":ab,ti OR "myopathy, mitochondrial encephalopathy lactic acidosis stroke":ab,ti OR "mitochondrial encephalomyopathy, lactic acidosis, stroke like episodes":ab,ti OR "mitochondrial myopathy, encephalopathy, lactic acidosis, stroke like episodes":ab,ti OR "encephalomyopathy, mitochondrial, lactic acidosis, stroke like episodes":ab,ti ) AND (mutation/de ORvariant*:ab,ti OR polymorphism*:ab,ti OR ( (substit*:ab,ti OR insert*:ab,ti OR delet*:ab,ti) AND mtdna:ab,ti AND base:ab,ti )))ANDhuman/deAND(article/it OR clinical trial/it) |
| **WOS** | (AB=(Mitochondri*) AND ( AB=(MELAS) OR ALL=("Mitochondrial Myopathy, Lactic Acidosis, Stroke-Like Episode") OR ALL=("Myopathy, Mitochondrial-Encephalopathy-Lactic Acidosis-Stroke") OR ALL=("Mitochondrial Encephalomyopathy, Lactic Acidosis, and Stroke-Like Episodes") OR ALL=("Mitochondrial Myopathy, Encephalopathy, Lactic Acidosis, And Stroke-Like Episodes") OR ALL=("Encephalomyopathy, Mitochondrial, Lactic Acidosis, and Stroke-Like Episodes")) AND (((ALL=(Substit*) OR ALL=(Insert*) OR ALL=(Delet*)) AND (ALL=(mtDNA) AND ALL=(Base))) OR (ALL=(Mutation) OR ALL=(Variant*) OR ALL=(Polymorphism*)))) AND LA=("English") AND DT=("Article") |

**Table S2. Characteristics of included studies**

| **PubMed ID** | **First author** | **Year** | **No. MELAS** | **No. mt.3243 A>G** | **No. MELAS (symptoms)** | **No. patients with symptoms** | | **No. MELAS & SLLs** | **No. with regions affected** | |
| --- | --- | --- | --- | --- | --- | --- | --- | --- | --- | --- |
| 1674297 | Hammand SR[8] | 1991 | 8 | 7 | - | - | | - | - | |
| 1586140 | Ciafaloni E[9] | 1992 | 23 | 21 | 23 | seizure | 23 | - | - | |
|  |  |  |  |  |  | blindness | 17 |  |  |  |
|  |  |  |  |  |  | ataxia | 3 |  |  |  |
|  |  |  |  |  |  | lactate elevated | 22 |  |  |  |
|  |  |  |  |  |  | MERRF | 20 |  |  |  |
|  |  |  |  |  |  | stroke | 23 |  |  |  |
| 9302261 | Hofmann S[10] | 1997 | 9 | 9 | - | - | | - | - | |
| 17276742 | Qi Y[11] | 2007 | - | - | 25 | seizure | 18 | - | - | |
|  |  |  |  |  |  | stroke | 19 |  |  |  |
|  |  |  |  |  |  | MERRF | 18 |  |  |  |
| 18306232 | Janssen AJM[12] | 2008 | - | - | 24 | lactate elevated | 20 | - | - | |
|  |  |  |  |  |  | seizure | 24 |  |  |  |
|  |  |  |  |  |  | hearing loss | 12 |  |  |  |
|  |  |  |  |  |  | MERRF | 15 |  |  |  |
| 22094475 | Kaufmann P[13] | 2011 | - | - | 31 | seizure | 31 | - | - | |
|  |  |  |  |  |  | stroke | 31 |  |  |  |
|  |  |  |  |  |  | hearing loss | 22 |  |  |  |
|  |  |  |  |  |  | diabetes | 12 |  |  |  |
|  |  |  |  |  |  | blindness | 14 |  |  |  |
| 3419020 | Galnares-Olalde JA[14] | 2021 | 6 | 4 | 6 | stroke  seizure  blindness  migraine  hearing loss  myopathy  MERRF | 6  6  4  3  1  1  5 | 6 | temporal | 6 |
|  |  |  |  |  |  |  |  |  | parietal | 5 |
|  |  |  |  |  |  |  |  |  | occipital | 4 |
|  |  |  |  |  |  |  |  |  | basal ganglion | 2 |
| 33951347 | Gramegna LL[15] | 2021 | 6 | 3 | 6 | seizure  stroke  myopathy  migraine  hearing loss  diabetes | 6  6  4  3  3  1 | 6 | temporal | 1 |
|  |  |  |  |  |  |  |  |  | parietal | 3 |
|  |  |  |  |  |  |  |  |  | occipital | 3 |
|  |  |  |  |  |  |  |  |  | basal ganglion | 2 |
| 34927673 | Ng YS[16] | 2022 | 111 | 72 | - | - | | - | - | |
| 36254078 | Chang X[2] | 2022 | - | - | 10 | seizure  migraine  diabetes  hearing loss  myopathy  ataxia  MERRF | 8  6  6  4  4  3  8 | 10 | temporal | 10 |
|  |  |  |  |  |  |  |  |  | parietal | 5 |
|  |  |  |  |  |  |  |  |  | occipital | 4 |
| 36202533 | Di Toro A[17] | 2022 | - | - | 14 | diabetes | 14 | - | - | |
|  |  |  |  |  |  | hearing loss | 14 |  |  |  |
| 37221696 | Wang W[1] | 2023 | 113 | 87 | 105 | seizure  migraine  blindness  hearing loss  lactate elevated  ataxia | 76  60  41  52  55  14 | 67 | parietal | 26 |
|  |  |  |  |  |  |  |  |  | temporal | 36 |
|  |  |  |  |  |  |  |  |  | occipital | 32 |
|  |  |  |  |  |  |  |  |  | basal ganglion | 3 |
| 39118480 | Zhao Y[3] | 2024 | 608 | 531 | 608 | seizure | 321 | - | - | |
|  |  |  |  |  |  | migraine | 162 |  |  |  |
|  |  |  |  |  |  | hearing loss | 126 |  |  |  |

**Table S3. Subgroup analysis of pooled estimates by onset age.**

|  | **Subgroup (No. of papers)** | **Pooled Proportion** | **Heterogeneity** | | |
| --- | --- | --- | --- | --- | --- |
|  |  |  | I^2^ | τ^2^ | p |
| **Genetic mutation** | | | | | |
| Pr. 3243 mutation | > 18 (3) | 0.74 (0.66, 0.81) | 0% | 0 | 0.081 |
| **Clinical symptoms** | | | | | |
| Hearing loss | > 18 (4) | 0.47 (0.39, 0.56) | 0% | 0 | 0.401 |
| Migraine | > 18 (4) | 0.57 (0.48, 0.65) | 0% | 0 | 0.966 |
| Myopathy | > 18 (3) | 0.41 (0.23, 0.62) | 0% | 0 | 0.196 |
| Seizure | ≤ 18 (3) | 0.76 (0.67, 0.82) | 94.10% | 2.98 | <0.001 |
|  | > 18 (4) | 0.84 (0.34, 0.98) | 0% | 0 | 0.060 |
| **Neuroimaging** | | | | | |
| Temporal | > 18 (3) | 0.98 (0.02, 0.99) | 92.09% | 20.54 | <0.001 |
| Occipital | > 18 (3) | 0.50 (0.30, 0.70) | 0% | 0 | 0.582 |
| Parietal | > 18 (3) | 0.59 (0.38, 0.77) | 0% | 0 | 0.336 |

**Table S4. Subgroup analysis of pooled estimates by gender.**

|  | **Subgroup (No. of papers)** | **Pooled Proportion** | **Heterogeneity** | | |
| --- | --- | --- | --- | --- | --- |
|  |  |  | I^2^ | τ^2^ | p |
| **Genetic mutation** | | | | | |
| Pr. 3243 mutation | Female ≤ 50% (3) | 0.77 (0.54, 0.91) | 82.00% | 0.27 | 0.002 |
|  | Female > 50% (3) | 0.76 (0.55, 0.89) | 48.48% | 0.33 | 0.023 |
| **Clinical symptoms** | | | | | |
| Hearing loss | Female ≤ 50% (4) | 0.36 (0.22, 0.53) | 83.80% | 0.32 | <0.001 |
|  | Female > 50% (4) | 0.68 (0.23, 0.94) | 89.38% | 3.14 | <0.001 |
| MERRF | Female > 50% (3) | 0.77 (0.58, 0.89) | 29.37% | 0.18 | 0.130 |
| Migraine | Female ≤ 50% (4) | 0.45 (0.28, 0.63) | 84.75% | 0.34 | <0.001 |
| Seizure | Female ≤ 50% (5) | 0.70 (0.55, 0.81) | 73.82% | 0.23 | <0.001 |
|  | Female > 50% (4) | 0.99 (0.20, 0.99) | 91.72% | 13.24 | <0.001 |

**Table S5. Risk of bias for included studies**

| **PubMed ID** | | **1674297** | **First Author** | **Hammand SR** | **Publication Year** | | **1991** |
| --- | --- | --- | --- | --- | --- | --- | --- |
| **Assessment Criteria** | | | | | | **Risk of Bias Adjustment** | |
| 1. | The sample frame was appropriate to address the target population. | | | | | Low | |
| 2. | The study participants were sampled in an appropriate way. | | | | | Moderate | |
| 3. | The sample size was adequate. | | | | | High | |
| 4. | The study subjects and the setting were described in detail. | | | | | Moderate | |
| 5. | The data analysis was conducted with sufficient coverage of the identified sample. | | | | | Moderate | |
| 6. | Valid methods were used for the identification of the condition. | | | | | Low | |
| 7. | The condition was measured in a standard, reliable way for all participants. | | | | | Low | |
| 8. | There was appropriate statistical analysis. | | | | | High | |
| 9. | The response rate was adequate OR the low response rate was managed appropriately. | | | | | Low | |
| **Overall bias** |  | | | | | **High** | |
| **PubMed ID** | | **1586140** | **First Author** | **Ciafaloni E** | **Publication Year** | | **1992** |
| **Assessment Criteria** | | | | | | **Risk of Bias Adjustment** | |
| 1. | The sample frame was appropriate to address the target population. | | | | | Low | |
| 2. | The study participants were sampled in an appropriate way. | | | | | Moderate | |
| 3. | The sample size was adequate. | | | | | Moderate | |
| 4. | The study subjects and the setting were described in detail. | | | | | Low | |
| 5. | The data analysis was conducted with sufficient coverage of the identified sample. | | | | | Moderate | |
| 6. | Valid methods were used for the identification of the condition. | | | | | Low | |
| 7. | The condition was measured in a standard, reliable way for all participants. | | | | | Low | |
| 8. | There was appropriate statistical analysis. | | | | | Moderate | |
| 9. | The response rate was adequate OR the low response rate was managed appropriately. | | | | | Low | |
| **Overall bias** |  | | | | | **Moderate** | |
| **PubMed ID** | | **9302261** | **First Author** | **Hofmann S** | **Publication Year** | | **1997** |
| **Assessment Criteria** | | | | | | **Risk of Bias Adjustment** | |
| 1. | The sample frame was appropriate to address the target population. | | | | | Moderate | |
| 2. | The study participants were sampled in an appropriate way. | | | | | Moderate | |
| 3. | The sample size was adequate. | | | | | Low | |
| 4. | The study subjects and the setting were described in detail. | | | | | Moderate | |
| 5. | The data analysis was conducted with sufficient coverage of the identified sample. | | | | | Low | |
| 6. | Valid methods were used for the identification of the condition. | | | | | Low | |
| 7. | The condition was measured in a standard, reliable way for all participants. | | | | | Low | |
| 8. | There was appropriate statistical analysis. | | | | | Moderate | |
| 9. | The response rate was adequate OR the low response rate was managed appropriately. | | | | | Low | |
| **Overall bias** |  | | | | | **Moderate** | |
| **PubMed ID** | | **17276742** | **First Author** | **Qi Y** | **Publication Year** | | **2007** |
| **Assessment Criteria** | | | | | | **Risk of Bias Adjustment** | |
| 1. | The sample frame was appropriate to address the target population. | | | | | Moderate | |
| 2. | The study participants were sampled in an appropriate way. | | | | | Moderate | |
| 3. | The sample size was adequate. | | | | | Low | |
| 4. | The study subjects and the setting were described in detail. | | | | | Moderate | |
| 5. | The data analysis was conducted with sufficient coverage of the identified sample. | | | | | Low | |
| 6. | Valid methods were used for the identification of the condition. | | | | | Moderate | |
| 7. | The condition was measured in a standard, reliable way for all participants. | | | | | Low | |
| 8. | There was appropriate statistical analysis. | | | | | Moderate | |
| 9. | The response rate was adequate OR the low response rate was managed appropriately. | | | | | Low | |
| **Overall bias** |  | | | | | **High** | |
| **PubMed ID** | | **18306232** | **First Author** | **Janssen AJM** | **Publication Year** | | **2008** |
| **Assessment Criteria** | | | | | | **Risk of Bias Adjustment** | |
| 1. | The sample frame was appropriate to address the target population. | | | | | Moderate | |
| 2. | The study participants were sampled in an appropriate way. | | | | | Low | |
| 3. | The sample size was adequate. | | | | | Moderate | |
| 4. | The study subjects and the setting were described in detail. | | | | | Low | |
| 5. | The data analysis was conducted with sufficient coverage of the identified sample. | | | | | Low | |
| 6. | Valid methods were used for the identification of the condition. | | | | | Low | |
| 7. | The condition was measured in a standard, reliable way for all participants. | | | | | Low | |
| 8. | There was appropriate statistical analysis. | | | | | Low | |
| 9. | The response rate was adequate OR the low response rate was managed appropriately. | | | | | Low | |
| **Overall bias** |  | | | | | **Moderate** | |
| **PubMed ID** | | **22094475** | **First Author** | **Kaufmann P** | **Publication Year** | | **2011** |
| **Assessment Criteria** | | | | | | **Risk of Bias Adjustment** | |
| 1. | The sample frame was appropriate to address the target population. | | | | | Moderate | |
| 2. | The study participants were sampled in an appropriate way. | | | | | Low | |
| 3. | The sample size was adequate. | | | | | Low | |
| 4. | The study subjects and the setting were described in detail. | | | | | Low | |
| 5. | The data analysis was conducted with sufficient coverage of the identified sample. | | | | | Moderate | |
| 6. | Valid methods were used for the identification of the condition. | | | | | Low | |
| 7. | The condition was measured in a standard, reliable way for all participants. | | | | | Low | |
| 8. | There was appropriate statistical analysis. | | | | | Low | |
| 9. | The response rate was adequate OR the low response rate was managed appropriately. | | | | | Moderate | |
| **Overall bias** |  | | | | | **Moderate** | |
| **PubMed ID** | | **3419020** | **First Author** | **Galnares-Olalde JA** | **Publication Year** | | **2021** |
| **Assessment Criteria** | | | | | | **Risk of Bias Adjustment** | |
| 1. | The sample frame was appropriate to address the target population. | | | | | Moderate | |
| 2. | The study participants were sampled in an appropriate way. | | | | | Moderate | |
| 3. | The sample size was adequate. | | | | | High | |
| 4. | The study subjects and the setting were described in detail. | | | | | Low | |
| 5. | The data analysis was conducted with sufficient coverage of the identified sample. | | | | | Low | |
| 6. | Valid methods were used for the identification of the condition. | | | | | Low | |
| 7. | The condition was measured in a standard, reliable way for all participants. | | | | | Low | |
| 8. | There was appropriate statistical analysis. | | | | | High | |
| 9. | The response rate was adequate OR the low response rate was managed appropriately. | | | | | Low | |
| **Overall bias** |  | | | | | **High** | |
| **PubMed ID** | | **33951347** | **First Author** | **Gramegna LL** | **Publication Year** | | **2021** |
| **Assessment Criteria** | | | | | | **Risk of Bias Adjustment** | |
| 1. | The sample frame was appropriate to address the target population. | | | | | Low | |
| 2. | The study participants were sampled in an appropriate way. | | | | | Low | |
| 3. | The sample size was adequate. | | | | | Moderate | |
| 4. | The study subjects and the setting were described in detail. | | | | | Low | |
| 5. | The data analysis was conducted with sufficient coverage of the identified sample. | | | | | Moderate | |
| 6. | Valid methods were used for the identification of the condition. | | | | | Low | |
| 7. | The condition was measured in a standard, reliable way for all participants. | | | | | Low | |
| 8. | There was appropriate statistical analysis. | | | | | Low | |
| 9. | The response rate was adequate OR the low response rate was managed appropriately. | | | | | Low | |
| **Overall bias** |  | | | | | **Moderate** | |
| **PubMed ID** | | **34927673** | **First Author** | **Ng YS** | **Publication Year** | | **2022** |
| **Assessment Criteria** | | | | | | **Risk of Bias Adjustment** | |
| 1. | The sample frame was appropriate to address the target population. | | | | | Low | |
| 2. | The study participants were sampled in an appropriate way. | | | | | Low | |
| 3. | The sample size was adequate. | | | | | Low | |
| 4. | The study subjects and the setting were described in detail. | | | | | Low | |
| 5. | The data analysis was conducted with sufficient coverage of the identified sample. | | | | | Moderate | |
| 6. | Valid methods were used for the identification of the condition. | | | | | Low | |
| 7. | The condition was measured in a standard, reliable way for all participants. | | | | | Low | |
| 8. | There was appropriate statistical analysis. | | | | | Low | |
| 9. | The response rate was adequate OR the low response rate was managed appropriately. | | | | | Low | |
| **Overall bias** |  | | | | | **Low** | |
| **PubMed ID** | | **36254078** | **First Author** | **Chang X** | **Publication Year** | | **2022** |
| **Assessment Criteria** | | | | | | **Risk of Bias Adjustment** | |
| 1. | The sample frame was appropriate to address the target population. | | | | | Moderate | |
| 2. | The study participants were sampled in an appropriate way. | | | | | Low | |
| 3. | The sample size was adequate. | | | | | High | |
| 4. | The study subjects and the setting were described in detail. | | | | | Low | |
| 5. | The data analysis was conducted with sufficient coverage of the identified sample. | | | | | Low | |
| 6. | Valid methods were used for the identification of the condition. | | | | | Low | |
| 7. | The condition was measured in a standard, reliable way for all participants. | | | | | Low | |
| 8. | There was appropriate statistical analysis. | | | | | Low | |
| 9. | The response rate was adequate OR the low response rate was managed appropriately. | | | | | Low | |
| **Overall bias** |  | | | | | **High** | |
| **PubMed ID** | | **36202533** | **First Author** | **Di Toro A** | **Publication Year** | | **2022** |
| **Assessment Criteria** | | | | | | **Risk of Bias Adjustment** | |
| 1. | The sample frame was appropriate to address the target population. | | | | | Moderate | |
| 2. | The study participants were sampled in an appropriate way. | | | | | Low | |
| 3. | The sample size was adequate. | | | | | High | |
| 4. | The study subjects and the setting were described in detail. | | | | | Low | |
| 5. | The data analysis was conducted with sufficient coverage of the identified sample. | | | | | Low | |
| 6. | Valid methods were used for the identification of the condition. | | | | | Low | |
| 7. | The condition was measured in a standard, reliable way for all participants. | | | | | Low | |
| 8. | There was appropriate statistical analysis. | | | | | Moderate | |
| 9. | The response rate was adequate OR the low response rate was managed appropriately. | | | | | Low | |
| **Overall bias** |  | | | | | **High** | |
| **PubMed ID** | | **37221696** | **First Author** | **Wang W** | **Publication Year** | | **2023** |
| **Assessment Criteria** | | | | | | **Risk of Bias Adjustment** | |
| 1. | The sample frame was appropriate to address the target population. | | | | | Low | |
| 2. | The study participants were sampled in an appropriate way. | | | | | Low | |
| 3. | The sample size was adequate. | | | | | Low | |
| 4. | The study subjects and the setting were described in detail. | | | | | Low | |
| 5. | The data analysis was conducted with sufficient coverage of the identified sample. | | | | | Moderate | |
| 6. | Valid methods were used for the identification of the condition. | | | | | Low | |
| 7. | The condition was measured in a standard, reliable way for all participants. | | | | | Low | |
| 8. | There was appropriate statistical analysis. | | | | | Low | |
| 9. | The response rate was adequate OR the low response rate was managed appropriately. | | | | | Low | |
| **Overall bias** |  | | | | | **Low** | |
| **PubMed ID** | | **39118480** | **First Author** | **Zhao Y** | **Publication Year** | | **2024** |
| **Assessment Criteria** | | | | | | **Risk of Bias Adjustment** | |
| 1. | The sample frame was appropriate to address the target population. | | | | | Low | |
| 2. | The study participants were sampled in an appropriate way. | | | | | Moderate | |
| 3. | The sample size was adequate. | | | | | Low | |
| 4. | The study subjects and the setting were described in detail. | | | | | Low | |
| 5. | The data analysis was conducted with sufficient coverage of the identified sample. | | | | | Low | |
| 6. | Valid methods were used for the identification of the condition. | | | | | Low | |
| 7. | The condition was measured in a standard, reliable way for all participants. | | | | | Low | |
| 8. | There was appropriate statistical analysis. | | | | | Low | |
| 9. | The response rate was adequate OR the low response rate was managed appropriately. | | | | | Low | |
| **Overall bias** |  | | | | | **Low** | |

**Table S6. Shared gene analysis across the MELAS-related pathways**

|  | MNRR1_import.gene | MRNN1_degrad.gene | mitophagy.gene | lc3_autophage.gene | tRNA_mito.gene | pi3k.gene | ampk.gene | nfkb.gene | glutamate.gene | arginine.gene |
| --- | --- | --- | --- | --- | --- | --- | --- | --- | --- | --- |
| ACO2 | 1 | 1 | 0 | 0 | 0 | 0 | 0 | 0 | 0 | 0 |
| AKT1 | 0 | 0 | 0 | 1 | 0 | 1 | 1 | 0 | 0 | 0 |
| AKT1S1 | 0 | 0 | 0 | 1 | 0 | 0 | 1 | 0 | 0 | 0 |
| AKT2 | 0 | 0 | 0 | 1 | 0 | 1 | 1 | 0 | 0 | 0 |
| AKT3 | 0 | 0 | 0 | 1 | 0 | 1 | 1 | 0 | 0 | 0 |
| ARG2 | 0 | 1 | 0 | 0 | 0 | 0 | 0 | 0 | 0 | 1 |
| ATG12 | 0 | 0 | 1 | 1 | 0 | 0 | 0 | 0 | 0 | 0 |
| ATG5 | 0 | 0 | 1 | 1 | 0 | 0 | 0 | 0 | 0 | 0 |
| ATG9A | 0 | 0 | 1 | 1 | 0 | 0 | 0 | 0 | 0 | 0 |
| ATP5F1A | 1 | 1 | 0 | 0 | 0 | 0 | 0 | 0 | 0 | 0 |
| ATP5F1B | 1 | 1 | 0 | 0 | 0 | 0 | 0 | 0 | 0 | 0 |
| BAD | 0 | 0 | 0 | 1 | 0 | 1 | 0 | 0 | 0 | 0 |
| BCL2 | 0 | 0 | 0 | 1 | 0 | 1 | 0 | 1 | 0 | 0 |
| BCL2L1 | 0 | 0 | 0 | 1 | 0 | 1 | 0 | 1 | 0 | 0 |
| CAMKK2 | 0 | 0 | 0 | 1 | 0 | 0 | 1 | 0 | 0 | 0 |
| CCND1 | 0 | 0 | 0 | 0 | 0 | 1 | 1 | 0 | 0 | 0 |
| CFLAR | 0 | 0 | 0 | 1 | 0 | 0 | 0 | 1 | 0 | 0 |
| CHCHD2 | 1 | 1 | 0 | 0 | 0 | 0 | 0 | 0 | 0 | 0 |
| CHUK | 0 | 0 | 0 | 0 | 0 | 1 | 0 | 1 | 0 | 0 |
| CREB1 | 0 | 0 | 0 | 0 | 0 | 1 | 1 | 0 | 0 | 0 |
| CREB3 | 0 | 0 | 0 | 0 | 0 | 1 | 1 | 0 | 0 | 0 |
| CREB3L1 | 0 | 0 | 0 | 0 | 0 | 1 | 1 | 0 | 0 | 0 |
| CREB3L2 | 0 | 0 | 0 | 0 | 0 | 1 | 1 | 0 | 0 | 0 |
| CREB3L3 | 0 | 0 | 0 | 0 | 0 | 1 | 1 | 0 | 0 | 0 |
| CREB3L4 | 0 | 0 | 0 | 0 | 0 | 1 | 1 | 0 | 0 | 0 |
| CREB5 | 0 | 0 | 0 | 0 | 0 | 1 | 1 | 0 | 0 | 0 |
| CRTC2 | 0 | 0 | 0 | 0 | 0 | 1 | 1 | 0 | 0 | 0 |
| CS | 1 | 1 | 0 | 0 | 0 | 0 | 0 | 0 | 0 | 0 |
| CSNK2A1 | 0 | 0 | 1 | 0 | 0 | 0 | 0 | 1 | 0 | 0 |
| CSNK2A2 | 0 | 0 | 1 | 0 | 0 | 0 | 0 | 1 | 0 | 0 |
| CSNK2B | 0 | 0 | 1 | 0 | 0 | 0 | 0 | 1 | 0 | 0 |
| DDIT4 | 0 | 0 | 0 | 1 | 0 | 1 | 0 | 0 | 0 | 0 |
| EIF4EBP1 | 0 | 0 | 0 | 0 | 0 | 1 | 1 | 0 | 0 | 0 |
| FOXO3 | 0 | 0 | 0 | 0 | 0 | 1 | 1 | 0 | 0 | 0 |
| G6PC1 | 0 | 0 | 0 | 0 | 0 | 1 | 1 | 0 | 0 | 0 |
| G6PC2 | 0 | 0 | 0 | 0 | 0 | 1 | 1 | 0 | 0 | 0 |
| G6PC3 | 0 | 0 | 0 | 0 | 0 | 1 | 1 | 0 | 0 | 0 |
| GLS | 0 | 0 | 0 | 0 | 0 | 0 | 0 | 0 | 1 | 1 |
| GLS2 | 0 | 0 | 0 | 0 | 0 | 0 | 0 | 0 | 1 | 1 |
| GLUD1 | 0 | 1 | 0 | 0 | 0 | 0 | 0 | 0 | 0 | 1 |
| GLUL | 0 | 0 | 0 | 0 | 0 | 0 | 0 | 0 | 1 | 1 |
| GNB1 | 0 | 0 | 0 | 0 | 0 | 1 | 0 | 0 | 1 | 0 |
| GNB2 | 0 | 0 | 0 | 0 | 0 | 1 | 0 | 0 | 1 | 0 |
| GNB3 | 0 | 0 | 0 | 0 | 0 | 1 | 0 | 0 | 1 | 0 |
| GNB4 | 0 | 0 | 0 | 0 | 0 | 1 | 0 | 0 | 1 | 0 |
| GNB5 | 0 | 0 | 0 | 0 | 0 | 1 | 0 | 0 | 1 | 0 |
| GNG10 | 0 | 0 | 0 | 0 | 0 | 1 | 0 | 0 | 1 | 0 |
| GNG11 | 0 | 0 | 0 | 0 | 0 | 1 | 0 | 0 | 1 | 0 |
| GNG12 | 0 | 0 | 0 | 0 | 0 | 1 | 0 | 0 | 1 | 0 |
| GNG13 | 0 | 0 | 0 | 0 | 0 | 1 | 0 | 0 | 1 | 0 |
| GNG2 | 0 | 0 | 0 | 0 | 0 | 1 | 0 | 0 | 1 | 0 |
| GNG3 | 0 | 0 | 0 | 0 | 0 | 1 | 0 | 0 | 1 | 0 |
| GNG4 | 0 | 0 | 0 | 0 | 0 | 1 | 0 | 0 | 1 | 0 |
| GNG5 | 0 | 0 | 0 | 0 | 0 | 1 | 0 | 0 | 1 | 0 |
| GNG7 | 0 | 0 | 0 | 0 | 0 | 1 | 0 | 0 | 1 | 0 |
| GNG8 | 0 | 0 | 0 | 0 | 0 | 1 | 0 | 0 | 1 | 0 |
| GNGT1 | 0 | 0 | 0 | 0 | 0 | 1 | 0 | 0 | 1 | 0 |
| GNGT2 | 0 | 0 | 0 | 0 | 0 | 1 | 0 | 0 | 1 | 0 |
| GYS1 | 0 | 0 | 0 | 0 | 0 | 1 | 1 | 0 | 0 | 0 |
| GYS2 | 0 | 0 | 0 | 0 | 0 | 1 | 1 | 0 | 0 | 0 |
| HRAS | 0 | 0 | 0 | 1 | 0 | 1 | 0 | 0 | 0 | 0 |
| HSD17B10 | 0 | 1 | 0 | 0 | 1 | 0 | 0 | 0 | 0 | 0 |
| HSPA9 | 1 | 1 | 0 | 0 | 0 | 0 | 0 | 0 | 0 | 0 |
| HSPD1 | 1 | 1 | 0 | 0 | 0 | 0 | 0 | 0 | 0 | 0 |
| IGF1 | 0 | 0 | 0 | 0 | 0 | 1 | 1 | 0 | 0 | 0 |
| IGF1R | 0 | 0 | 0 | 1 | 0 | 1 | 1 | 0 | 0 | 0 |
| IGH | 0 | 0 | 0 | 0 | 0 | 1 | 0 | 1 | 0 | 0 |
| IKBKB | 0 | 0 | 0 | 0 | 0 | 1 | 0 | 1 | 0 | 0 |
| IKBKG | 0 | 0 | 0 | 0 | 0 | 1 | 0 | 1 | 0 | 0 |
| INS | 0 | 0 | 0 | 1 | 0 | 1 | 1 | 0 | 0 | 0 |
| INSR | 0 | 0 | 0 | 0 | 0 | 1 | 1 | 0 | 0 | 0 |
| IRS1 | 0 | 0 | 0 | 1 | 0 | 1 | 1 | 0 | 0 | 0 |
| IRS2 | 0 | 0 | 0 | 1 | 0 | 0 | 1 | 0 | 0 | 0 |
| IRS4 | 0 | 0 | 0 | 1 | 0 | 0 | 1 | 0 | 0 | 0 |
| ITPR1 | 0 | 0 | 0 | 1 | 0 | 0 | 0 | 0 | 1 | 0 |
| KRAS | 0 | 0 | 0 | 1 | 0 | 1 | 0 | 0 | 0 | 0 |
| LDHD | 1 | 1 | 0 | 0 | 0 | 0 | 0 | 0 | 0 | 0 |
| MAP1LC3A | 0 | 0 | 1 | 1 | 0 | 0 | 0 | 0 | 0 | 0 |
| MAP1LC3B | 0 | 0 | 1 | 1 | 0 | 0 | 0 | 0 | 0 | 0 |
| MAP2K1 | 0 | 0 | 0 | 1 | 0 | 1 | 0 | 0 | 0 | 0 |
| MAP2K2 | 0 | 0 | 0 | 1 | 0 | 1 | 0 | 0 | 0 | 0 |
| MAP3K7 | 0 | 0 | 0 | 1 | 0 | 0 | 1 | 1 | 0 | 0 |
| MAPK1 | 0 | 0 | 0 | 1 | 0 | 1 | 0 | 0 | 1 | 0 |
| MAPK3 | 0 | 0 | 0 | 1 | 0 | 1 | 0 | 0 | 1 | 0 |
| MLST8 | 0 | 0 | 0 | 1 | 0 | 1 | 0 | 0 | 0 | 0 |
| MTOR | 0 | 0 | 0 | 1 | 0 | 1 | 1 | 0 | 0 | 0 |
| NFKB1 | 0 | 0 | 0 | 0 | 0 | 1 | 0 | 1 | 0 | 0 |
| NOS3 | 0 | 0 | 0 | 0 | 0 | 1 | 0 | 0 | 0 | 1 |
| NRAS | 0 | 0 | 0 | 1 | 0 | 1 | 0 | 0 | 0 | 0 |
| OPTN | 0 | 0 | 1 | 1 | 0 | 0 | 0 | 0 | 0 | 0 |
| OTC | 1 | 0 | 0 | 0 | 0 | 0 | 0 | 0 | 0 | 1 |
| P3R3URF-PIK3R3 | 0 | 0 | 0 | 1 | 0 | 1 | 1 | 0 | 0 | 0 |
| PCK1 | 0 | 0 | 0 | 0 | 0 | 1 | 1 | 0 | 0 | 0 |
| PCK2 | 0 | 0 | 0 | 0 | 0 | 1 | 1 | 0 | 0 | 0 |
| PDPK1 | 0 | 0 | 0 | 1 | 0 | 1 | 1 | 0 | 0 | 0 |
| PIK3CA | 0 | 0 | 0 | 1 | 0 | 1 | 1 | 0 | 0 | 0 |
| PIK3CB | 0 | 0 | 0 | 1 | 0 | 1 | 1 | 0 | 0 | 0 |
| PIK3CD | 0 | 0 | 0 | 1 | 0 | 1 | 1 | 0 | 0 | 0 |
| PIK3R1 | 0 | 0 | 0 | 1 | 0 | 1 | 1 | 0 | 0 | 0 |
| PIK3R2 | 0 | 0 | 0 | 1 | 0 | 1 | 1 | 0 | 0 | 0 |
| PIK3R3 | 0 | 0 | 0 | 1 | 0 | 1 | 1 | 0 | 0 | 0 |
| PMPCA | 1 | 1 | 0 | 0 | 0 | 0 | 0 | 0 | 0 | 0 |
| PPP2CA | 0 | 0 | 0 | 1 | 0 | 1 | 1 | 0 | 0 | 0 |
| PPP2CB | 0 | 0 | 0 | 1 | 0 | 1 | 1 | 0 | 0 | 0 |
| PPP2R1A | 0 | 0 | 0 | 0 | 0 | 1 | 1 | 0 | 0 | 0 |
| PPP2R1B | 0 | 0 | 0 | 0 | 0 | 1 | 1 | 0 | 0 | 0 |
| PPP2R2A | 0 | 0 | 0 | 0 | 0 | 1 | 1 | 0 | 0 | 0 |
| PPP2R2B | 0 | 0 | 0 | 0 | 0 | 1 | 1 | 0 | 0 | 0 |
| PPP2R2C | 0 | 0 | 0 | 0 | 0 | 1 | 1 | 0 | 0 | 0 |
| PPP2R2D | 0 | 0 | 0 | 0 | 0 | 1 | 1 | 0 | 0 | 0 |
| PPP2R3A | 0 | 0 | 0 | 0 | 0 | 1 | 1 | 0 | 0 | 0 |
| PPP2R3B | 0 | 0 | 0 | 0 | 0 | 1 | 1 | 0 | 0 | 0 |
| PPP2R3C | 0 | 0 | 0 | 0 | 0 | 1 | 1 | 0 | 0 | 0 |
| PPP2R5A | 0 | 0 | 0 | 0 | 0 | 1 | 1 | 0 | 0 | 0 |
| PPP2R5B | 0 | 0 | 0 | 0 | 0 | 1 | 1 | 0 | 0 | 0 |
| PPP2R5C | 0 | 0 | 0 | 0 | 0 | 1 | 1 | 0 | 0 | 0 |
| PPP2R5D | 0 | 0 | 0 | 0 | 0 | 1 | 1 | 0 | 0 | 0 |
| PPP2R5E | 0 | 0 | 0 | 0 | 0 | 1 | 1 | 0 | 0 | 0 |
| PRKAA1 | 0 | 0 | 0 | 1 | 0 | 1 | 1 | 0 | 0 | 0 |
| PRKAA2 | 0 | 0 | 0 | 1 | 0 | 1 | 1 | 0 | 0 | 0 |
| PRKACA | 0 | 1 | 0 | 1 | 0 | 0 | 0 | 0 | 1 | 0 |
| PRKACB | 0 | 0 | 0 | 1 | 0 | 0 | 0 | 0 | 1 | 0 |
| PRKACG | 0 | 0 | 0 | 1 | 0 | 0 | 0 | 0 | 1 | 0 |
| PRKCA | 0 | 0 | 0 | 0 | 0 | 1 | 0 | 0 | 1 | 0 |
| PRKCB | 0 | 0 | 0 | 0 | 0 | 0 | 0 | 1 | 1 | 0 |
| PRKCQ | 0 | 0 | 0 | 1 | 0 | 0 | 0 | 1 | 0 | 0 |
| PTEN | 0 | 0 | 0 | 1 | 0 | 1 | 0 | 0 | 0 | 0 |
| RAB8A | 0 | 0 | 0 | 1 | 0 | 0 | 1 | 0 | 0 | 0 |
| RAF1 | 0 | 0 | 0 | 1 | 0 | 1 | 0 | 0 | 0 | 0 |
| RELA | 0 | 0 | 0 | 0 | 0 | 1 | 0 | 1 | 0 | 0 |
| RHEB | 0 | 0 | 0 | 1 | 0 | 1 | 1 | 0 | 0 | 0 |
| RPS27A | 0 | 0 | 1 | 1 | 0 | 0 | 0 | 0 | 0 | 0 |
| RPS6KB1 | 0 | 0 | 0 | 1 | 0 | 1 | 1 | 0 | 0 | 0 |
| RPS6KB2 | 0 | 0 | 0 | 1 | 0 | 1 | 1 | 0 | 0 | 0 |
| RPTOR | 0 | 0 | 0 | 1 | 0 | 1 | 1 | 0 | 0 | 0 |
| SLC25A6 | 1 | 1 | 0 | 0 | 0 | 0 | 0 | 0 | 0 | 0 |
| SQSTM1 | 0 | 0 | 1 | 1 | 0 | 0 | 0 | 0 | 0 | 0 |
| STK11 | 0 | 0 | 0 | 1 | 0 | 1 | 1 | 0 | 0 | 0 |
| SYK | 0 | 0 | 0 | 0 | 0 | 1 | 0 | 1 | 0 | 0 |
| TBK1 | 0 | 0 | 1 | 1 | 0 | 0 | 0 | 0 | 0 | 0 |
| TIMM10 | 1 | 1 | 0 | 0 | 0 | 0 | 0 | 0 | 0 | 0 |
| TIMM17A | 1 | 1 | 0 | 0 | 0 | 0 | 0 | 0 | 0 | 0 |
| TIMM22 | 1 | 1 | 0 | 0 | 0 | 0 | 0 | 0 | 0 | 0 |
| TIMM9 | 1 | 1 | 0 | 0 | 0 | 0 | 0 | 0 | 0 | 0 |
| TLR4 | 0 | 0 | 0 | 0 | 0 | 1 | 0 | 1 | 0 | 0 |
| TOMM20 | 1 | 0 | 1 | 0 | 0 | 0 | 0 | 0 | 0 | 0 |
| TOMM22 | 1 | 0 | 1 | 0 | 0 | 0 | 0 | 0 | 0 | 0 |
| TOMM40 | 1 | 0 | 1 | 0 | 0 | 0 | 0 | 0 | 0 | 0 |
| TOMM5 | 1 | 0 | 1 | 0 | 0 | 0 | 0 | 0 | 0 | 0 |
| TOMM6 | 1 | 0 | 1 | 0 | 0 | 0 | 0 | 0 | 0 | 0 |
| TOMM7 | 1 | 0 | 1 | 0 | 0 | 0 | 0 | 0 | 0 | 0 |
| TOMM70 | 1 | 0 | 1 | 0 | 0 | 0 | 0 | 0 | 0 | 0 |
| TRAF6 | 0 | 0 | 0 | 1 | 0 | 0 | 0 | 1 | 0 | 0 |
| TSC1 | 0 | 0 | 0 | 1 | 0 | 1 | 1 | 0 | 0 | 0 |
| TSC2 | 0 | 0 | 0 | 1 | 0 | 1 | 1 | 0 | 0 | 0 |
| UBA52 | 0 | 0 | 1 | 1 | 0 | 0 | 0 | 0 | 0 | 0 |
| UBB | 0 | 0 | 1 | 1 | 0 | 0 | 0 | 0 | 0 | 0 |
| UBC | 0 | 0 | 1 | 1 | 0 | 0 | 0 | 0 | 0 | 0 |
| ULK1 | 0 | 0 | 1 | 1 | 0 | 0 | 1 | 0 | 0 | 0 |
| VDAC1 | 1 | 0 | 1 | 0 | 0 | 0 | 0 | 0 | 0 | 0 |
